# A human monoclonal antibody isolated from Japanese encephalitis virus vaccine-vaccinated volunteer neutralizing various flaviviruses

**DOI:** 10.3389/fmicb.2024.1508923

**Published:** 2024-12-23

**Authors:** Dong Chen, Jiayi Zhang, Yusha Liu, Jiayang Zhu, Jie Chen, Hongxia Ni, Jinsheng Wen

**Affiliations:** ^1^School of Basic Medical Sciences, Health Science Center, Ningbo University, Ningbo, China; ^2^Wenzhou Central Blood Station, Wenzhou, China; ^3^Key Laboratory of Laboratory Medicine, Ministry of Education, Zhejiang Provincial Key Laboratory of Medical Genetics, College of Laboratory Medicine and Life Sciences, Wenzhou Medical University, Wenzhou, China; ^4^Ningbo Municipal Center for Disease Control and Prevention, Ningbo, China

**Keywords:** Japanese encephalitis virus, Zika virus, dengue virus, flavivirus, monoclonal antibody, neutralization, mouse, molecular docking

## Abstract

**Introduction:**

Japanese encephalitis virus (JEV) and Zika virus (ZIKV) are prevalent in over 80 countries or territories worldwide, causing hundreds of thousands of cases annually. But currently there is a lack of specific antiviral agents and effective vaccines.

**Methods:**

In the present study, to identify human neutralizing monoclonal antibody (mAb) against JEV or/and ZIKV, we isolated ZIKV-E protein-binding B cells from the peripheral venous blood of a healthy volunteer who had received the JEV live-attenuated vaccine and performed 10× Genomics transcriptome sequencing and BCR sequencing analysis, we then obtained the V region amino acid sequences of a novel mAb LZY3412. We expressed mAb LZY3412 and evaluated its ability to bind to E proteins of dengue virus, JEV and ZIKV, neutralize JEV and ZIKV infections *in vitro*, protect mice against lethal JEV or ZIKV attack. The epitopes on E proteins of JEV/ZIKV recognized by mAb LZY3412 were analyzed using molecular docking and constructing E protein mutants.

**Results:**

Our results show that recombinant mAb LZY3412 has high-affinity with the E proteins of three viruses, with the kinetically derived binding affinity (KD) values of 440 and 482.5 nM against JEV-E protein and ZIKV-E protein, respectively; recombinant mAb LZY3412 can efficiently neutralize JEV and ZIKV infections *in vitro*, with the NT_50_ values of 19.9 ng/mL and 631 ng/mL, respectively; application of recombinant mAb LZY3412 can significantly improve the percentage survival and reduce the serum viral loads of neonatal mice infected with JEV or ZIKV. Finally, two amino acid residues (Ala399 and Gly400) located in EDIII of JEV-E protein were potentially recognized by LZY3412 whereas two amino acid residues (Met15 and Thr406) out of EDIII of ZIKV-E proteins were recognized by LZY3412.

**Discussion:**

Considering mAb LZY3412 has neutralizing effects on various flaviviruses, this mAb is expected to be developed as therapeutic or preventive drug for these flaviviruses, and can also be used to guide the development of vaccines against these flaviviruses.

## Introduction

Flaviviruses are positive-sense, single-stranded RNA viruses that include dengue virus (DV, classified into four serotypes: D1V, D2V, D3V, and D4V), Japanese encephalitis virus (JEV), West Nile virus (WNV), and Zika virus (ZIKV), etc. (Kuno et al., 1998). JEV is mainly prevalent in 24 countries/territories in East Asia, Southeast Asia, Western Pacific, and South Asia (Centers for Disease Control and Prevention [CDC], 2013). JEV infection usually causes mild symptoms, but approximately 70,000 cases annually may progress to encephalitis, exhibiting a mortality rate of up to 30% (Campbell et al., 2011; Centers for Disease Control and Prevention [CDC], 2013). ZIKV was first discovered in 1947 and reported to cause a limited number of infections in the following decades of years. After more than half a century, ZIKV led to an outbreak in Brazil in 2015 and was later declared as a global health emergency by World Health Organization (WHO). It was estimated that there were 0.4–1.5 million cases of ZIKV infection globally from 2015 to 2017 (Ferraris et al., 2019). In addition to causing mild symptoms such as fever, ZIKV infection is also associated with severe diseases like Guillain–Barré syndrome and fetal birth defects (particularly microcephaly) (Li et al., 2016; Mlakar et al., 2016; Monsalve et al., 2017). Up to now, ZIKV has spread to over 80 countries worldwide (WHO, 2016) and poses a severe threat to human health (Santos et al., 2023). It should be emphasized that JEV and ZIKV co-circulate in many countries/territories, such as China, Japan, India, Southeast Asian countries, and countries in Western Pacific region (Kutsuna et al., 2014; Zhang et al., 2016; WHO, 2016; Quyen et al., 2017; Khaiboullina et al., 2018; Ruchusatsawat et al., 2019). JEV has approved vaccines including inactivated Vero cell-cultured viruses, inactivated mouse brain-cultured viruses, and live-attenuated JEV vaccine SA14-14-2 (Gao et al., 2014). However, there are no approved vaccines or antiviral agents against ZIKV.

The mature flaviviruses are spherical enveloped particles (Kostyuchenko et al., 2016; Sirohi et al., 2016). The RNA genome complexed with capsid proteins forms the core of the particle which is surrounded by a lipid bilayer membrane. The genome of flavivirus encodes three structural proteins composed of capsid (C), pre-membrane/membrane (prM/M), and Envelope (E) proteins. The E protein mediates the entry of flavivirus into susceptible cells and represents the critical antigen for inducing the production of neutralizing antibodies (Abs). The ectodomain of E protein consists of three domains – DI, DII, and DIII (Modis et al., 2003). E protein DI (EDI) is a central β-barrel domain that links E protein DII (EDII) and E protein DIII (EDIII). EDII is an elongated domain containing a fusion loop at its tip. EDIII contains the receptor-binding site and mediates virus attachment, and is also important for virus-endosomal membrane fusion (Modis et al., 2003). After binding to receptors, flavivirus particles are endocytosed and the acidic environment induces virus-endosomal membrane fusion, thereby releasing the viral genome into the cytosol (Rodenhuis-Zybert et al., 2011).

In recent years, especially since the global outbreak of the Zika virus in 2015, scientists have identified several human neutralizing monoclonal antibodies (mAbs) from B cells of individuals recovered from ZIKV infection (Sapparapu et al., 2016; Stettler et al., 2016; Wang et al., 2016; Hasan et al., 2017; Magnani et al., 2017; Robbiani et al., 2017; Yu et al., 2017; Bailey et al., 2019; Collins et al., 2019; Long et al., 2019; Niu et al., 2019; Dussupt et al., 2020; Sevvana et al., 2020; Zhang et al., 2020; Graham et al., 2021; Franca et al., 2022; Adams et al., 2023). Of note, some human ZIKV-neutralizing mAbs have the ability to cross-neutralize DENV infection simultaneously (Robbiani et al., 2017; Yu et al., 2017; Niu et al., 2019; Dussupt et al., 2020; Zhang et al., 2020; Zhao et al., 2020). But whether these human mAbs have cross-neutralizing effects on JEV infection has not been tested or identified yet. In comparison, a limited number of JEV-specific mouse mAbs with protective activity have been identified (Zhang et al., 1989; Fernandez et al., 2018; Qiu et al., 2018; Calvert et al., 2019). Of note, a humanized mAb (B2) engineered from a parental mAb that was isolated from a JEV-immunized chimpanzee could protect mice against JEV challenge (Goncalvez et al., 2008). In general, the number of human neutralizing mAbs against JEV is very limited (Fernandez et al., 2018; Ozawa et al., 2018). Notwithstanding these data, no study has comprehensively profiled the inhibitory activity of anti-JEV mAbs against other flaviviruses *in vitro* and *in vivo*, and no fully human anti-JEV mAbs with cross-neutralization against ZIKV have been described.

In order to screen out human mAb that has neutralizing activity against both JEV and ZIKV infections, in the present study, we isolated antigen-specific B cells from one healthy volunteer who had received the JEV live-attenuated vaccine and identified a cross-reactive human mAb LZY3412 through single-cell sequencing. mAb LZY3412 bound to the E proteins of DV, JEV and ZIKV with high-affinity, and exhibited potent neutralizing activity against JEV and ZIKV infections *in vitro* and *in vivo*. Therefore, human mAb LZY3412 may has preventive/therapeutic potential for JEV and ZIKV infections.

## Materials and methods

### Ethics statement

After receiving the informed consent form, the peripheral venous blood was drawn from one healthy volunteer (22-year-old individual who had been vaccinated with live-attenuated JEV vaccine SA14-14-2, once each at 8 months and 2 years of age). This study was approved by the ethical committee of Ningbo University (no. NBU-2020-002).

Six-week-old male and female wild-type (WT) C57BL/6 mice were purchased from the Animal Model Research Center at Nanjing University, China, and were maintained and bred in the Animal Center of Ningbo University. The experiments were performed in strict accordance with the animal welfare act and public health service policy on humane care and use of laboratory animals and were approved by the Animal Ethics Committee of Ningbo University (no. 2020–55). Sample sizes were estimated on the basis of our previous studies. Animal experiments were randomized but not blinded.

### Cell lines, viruses and reagents

Vero cell was purchased from American Type Culture Center (ATCC) and cultured in RPMI-1640 medium supplemented with 10% fetal bovine serum (FBS) and 1% penicillin/streptomycin. HEK-293 T cell was obtained from ATCC and cultured in MEM medium supplemented with 10% FBS and 1% penicillin/streptomycin. Both JEV (strain ZJ14-52, GenBank accession no: MK558811) and ZIKV (strain Zhejiang04, GenBank accession no: KX117076.1) were propagated in Vero cells and the viral stocks were tittered using plaque-forming assay (PFA) as described below. The viral titer was expressed as plaque-forming unit (PFU)/ml. HRP-conjugated goat anti-human IgG H chain or L chain (k chain) mAb, mouse anti-human IgG H chain mAb, and HRP-labeled goat anti-mouse IgG mAb were purchased from Proteintech (Chicago, United States). 3,3′,5,5′-Tetramethylbenzidine (TMB) was purchased from Macklin (Shanghai, China). Methyl cellulose M450, Isopropyl-*β*-d-Thiogalactoside (IPTG), Polyetherimide (PEI) transfection reagent, and Dithiothreitol (DTT) were purchased from Ourchem (Beijing, China), Solarbio (Beijing, China), GlpBio (California, United States), and Sigma-Aldrich (St. Louis, United States), respectively.

### Expression of viral proteins

The genes (optimized for *Escherichia coli* codon usage) encoding the extracellular domains or EDIIIs of E proteins of JEV (E406, aa 1–406; EDIII, aa 297–400; GenBank accession no: MK558811), ZIKV (E410, aa 1–410; EDIII aa 301–404; GenBank accession no: KX117076.1), D1V (E401, aa 1–401; EDIII aa 295–395; Hawaii strain, GenBank accession no: ACF49259), D2V (E401, aa 1–401; EDIII aa 295–395; New Guinea C strain, GenBank accession no: AIU47320), D3V (E399, aa 1–399; EDIII aa 293–393; H87 strain, GenBank accession no: P27915), and D4V (E401, aa 1–401; EDIII aa 295–395; H241 strain, GenBank accession no: Q58HT7) (Supplementary Table S1) were synthesized by GeneWiz (Suzhou, China) and cloned into the pET21a expression vectors, each featuring a 6 × His tag at the 3′ terminal of gene sequence. In addition, the genes encoding the mutants of JEV-E406 (JEV-E406^mut-1^, with Gln^258^Ala substitution; JEV-E406^mut-2^, with Ala^399^Arg substitution; JEV-E406^mut-3^, with Gly^400^Ala substitution; JEV-E406^mut-4^, with Gln^258^Ala, Ala^399^Arg and Gly^400^Ala substitutions) and ZIKV-E410 (ZIKV-E410^mut-1^, with Met^15^Ala substitution; ZIKV-E410^mut-2^, Arg^357^Ala substitution; ZIKV-E410^mut-3^, Thr^406^Ala substitution; ZIKV-E410^mut-4^, with Met^15^Ala, Arg^357^Ala and Thr^406^Ala substitutions) were also synthesized and cloned into pET21a. These recombinant plasmids were, respectively, transformed into *E. coli* BL21 strain, and protein expression was induced using IPTG at a final concentration of 1 mM. These recombinant proteins were purified using a Ni-NTA (TransGen Biotech, Beijing, China), followed by further concentration through a 10 kDa ultrafiltration tube (Millipore, Darmstadt, Germany). The protein purity was assessed by SDS-PAGE and the protein concentration was quantified using a BCA kit (TransGen Biotech).

### Isolation of ZIKV-E410-binding B cells

The peripheral blood mononuclear cells (PBMC) were isolated from the peripheral venous blood using the Ficoll according to the manufacturer’s instruction. Human B cells were isolated from PBMC by negative selection using a cocktail of biotinylated antibodies against other cells except B cells plus streptavidin-conjugated microbeads (Miltenyi, Germany). ZIKV-E410-binding B cells were isolated from human B cells by positive selection using ZIKV-E410-coated magnetic beads (1 μm in diameter). The isolated ZIKV-E410-binding B cells were sent to Bestop company (Tianjin, China) for performing 10 × Genomics transcriptome sequencing and BCR sequencing.

### Construction of recombinant plasmids encoding H or L chains of LZY3412

The gene sequences of the variable (V) region of BCR in over 6,000 ZIKV-E410-binding B cells were obtained. The V region sequences of the light (L) and heavy (H) chains of a novel mAb LZY3412 were chosen for further research. To express LZY3412, we designed two DNA sequences. One consists of a Kozak sequence, a signal peptide sequence, gene sequence encoding VH of LZY3412 and constant region of H chain (CH) of a previously reported mAb; the other is composed of a Kozak sequence, a signal peptide sequence, gene sequence encoding VL of LZY3412 and CL of a previously reported mAb. Two DNA sequences were optimized for *Homo sapiens* codon usage and cloned into the eukaryotic plasmid pCAGGS, resulting into two recombinant plasmids: pCAGGS-LZY3412H and pCAGGS-LZY3412L.

### Expression of recombinant LZY3412

HEK-293T cells were plated to cell culture plate (10 cm^2^) and incubated for 24 h in a 37°C/5%CO_2_ incubator. Next day, the cell supernatant was discarded and MEM medium without FBS was added to the cells. Four hours later, 20 μg of two recombinant plasmids (the weight ratio of pCAGGS-LZY3412H: pCAGGS-LZY3412L = 1.07:0.93) was diluted in PBS to reach 0.5 mL. Forty microgram (in 0.5 mL PBS) PEI was added to diluted recombinant plasmid and incubated for 20 min at room temperature. The PEI/plasmid mixture was added to cells and transfected for 4 h. After discarding the transfection reagent, complete MEM medium with 10% FBS was added to the cells and incubated in the incubator. The supernatant was collected at 48 and 96 h post transfection and filtered through a 0.22 μm filter, followed by purification using a Pierce protein A/G agarose column (Thermo Fisher). Subsequently, SDS-PAGE (in both reducing and non-reducing states) electrophoresis was performed, followed by WB experiment.

### ELISA

The capacity of recombinant mAb LZY3412 to bind live viruses (JEV and ZIKV) or recombinant proteins was first evaluated using ELISA. In brief, ELISA plate was coated with JEV viral particles (4 × 10^6^ PFU/ml, 100 μL/well), ZIKV viral particles (4 × 10^6^ PFU/ml, 100 μL/well), various recombinant proteins (20 μg/mL, 100 μL/well) for 1.5 h at 37°C. The plates are blocked with 5% skim milk/PBS for 1.5 h at 37°C. 3-fold serially diluted mAb LZY3412 (starting from 100 μg/mL) was added to the plate and incubated for 1.5 h at 37°C. Mouse anti-human IgG mAb (anti-heavy chain, 1:5000 dilution) was added to the plate and incubated for 1.5 h at 37°C. HRP-labeled goat anti-mouse IgG mAb (1:5000 dilution) was added to the plate and incubated for 1.5 h at 37°C. The plates were developed with fresh TMB solution for 15 min at room temperature. The OD450 value of each well was measured and analyzed using Prism software. Area under the curve (AUC) was used to compare the difference between two groups.

### Biolayer interferometry binding assay

Affinity determination between recombinant mAb LZY3412 and antigen JEV-E406 or ZIKV-E410 was performed using biolayer interferometry with a WeSPR One software (Liangzhun [Hangzhou] Scientific Instrument Co., Ltd.). Antigen JEV-E406 or ZIKV-E410 was coupled to LifeDisc^TM^ MetaSPR biosensors (Liangzhun [Hangzhou] Scientific Instrument Co., Ltd.). Unbound antigen was removed from the surfaces of the sensors by incubation in kinetics buffer (NTE buffer, 10 mM Tris–HCl pH 8.0, 120 mM NaCl and 1 mM EDTA). Sensors loaded with antigen were allowed to bind to different concentrations (40, 80, 160, and 320 μg/mL [0.267, 0.534, 1.068, and 2.136 μM, respectively]) of LZY3412. Association and dissociation profiles were fitted with WeSPR One software package assuming 1:1 binding model.

### Plaque reduction neutralization test

The ability of recombinant mAb LZY3412 to neutralize JEV or ZIKV infections *in vitro* was evaluated on Vero cells using a Plaque-Reduction Neutralization Test (PRNT). In brief, Vero cells were seeded in each well (1 × 10^5^ cells/well) of 24-well cell culture plate and incubated for 24 h in a 37°C/5% CO_2_ incubator. mAb LZY3412 was 3-fold serially diluted (starting from 100 μg/mL) in the well (240 μL/well) of 96-well culture plate, followed by addition of 50 μL of JEV or ZIKV solution (containing 50 PFU), and incubated for 1 h at 37°C. After discarding the supernatant of each well, the mixture of serum/virus was added to Vero cell and infected for 1 h at 37°C. After discarding the viral solution, the cells were overlaid with 1% Methyl cellulose/medium and incubated for 3 days in a 37°C/5% CO_2_ incubator. The cells were fixed with 4% paraformaldehyde for 1 h at room temperature and stained with 0.5% crystal violet solution for 10 min at room temperature. The plaques of each well were counted and the NT_50_ (the lowest antibody concentration that can reduce viral plaques by 50%) values were calculated.

### Mouse experiments

Ten PFU (in 10 μL PBS) of JEV or 1,000 PFU (in 10 μL PBS) of ZIKV was subcutaneously (s.c.) injected into 1-day-old neonatal mice. Two hours later, 1, 10 or 100 μg LZY3412 mAb (in 5 μL PBS) was injected s.c. into the mice. In one protocol, the mouse weights and mortality were recorded daily for 21 days or 28 days. In another protocol, 3 days after viral challenge, the mice were sacrificed and the mouse sera were harvested for viral load detection using PFA.

### Tissue viral loads detection

The titers of viral stock and the viral load in mouse sera were determined using PFA. Briefly, Vero cells are plated to each well (1 × 10^5^ cells/well) of 24-well cell culture plate and cultured for 24 h in a 37°C/5% CO_2_ incubator. After discarding the cell supernatant, 240 μL of serially diluted viral stock or mouse serum was added to cells and the plate was incubated for 1 h at 37°C. After discarding the samples, the cells were overlaid with 1% Methyl cellulose/medium and incubated for 3–5 days in a 37°C/5% CO_2_ incubator. The cells were fixed with 4% paraformaldehyde for 1 h at room temperature and stained with 0.5% crystal violet solution for 10 min at room temperature. The numbers of plaques in each well were counted and the viral titer in the viral stock or mouse serum was expressed as PFU/ml.

### Molecular docking

Based on the amino acid sequences of JEV-E protein, ZIKV-E protein, and LZY3412 heavy and light chains, the SWISS-MODEL program (Jumper et al., 2021) was used to perform homology modeling, thereby obtaining a three-dimensional structural model of antigen and Ab that can be used for subsequent molecular binding studies. In order to investigate the binding regions and interaction modes between JEV-E/ZIKV-E proteins and mAb LZY3412, a professional protein and protein DNA/RNA docking HDCOK program (Feng et al., 2022; Feng et al., 2021) was used for docking. The structure with the best docking score was selected as the standard result for subsequent interaction analysis. The docking score is calculated based on the ITScorePP or ITScorePR iterative scoring function. The more negative the docking score, the greater the possibility of binding and stronger interaction of the combined model. Considering that the docking score of protein–protein/RNA/DNA complexes in PDB is usually around −200 or better, we have defined a confidence score dependent on docking score based on experience to represent the binding possibility of two molecules as follows:

Confidence_score = 1.0/[1.0 + e0.02*(Docking_Score+150)].

Generally speaking, when the confidence score is above 0.7, two molecules are more likely to interact with each other; when the confidence score is between 0.5 and 0.7, these two molecules are likely to interact with each other; but when the confidence score is below 0.5, these two molecules are unlikely to bind to each other.

### Statistical analysis

All data were analyzed using Prism 8 software (GraphPad Software, La Jolla, CA, United States) and expressed as the means ± SEM. The comparison between group means and survival data were analyzed using the Mann–Whitney test and a log-rank test, respectively. *p* < 0.05 was considered statistically significant.

## Results

### The characteristics of a novel mAb LZY3412 identified from healthy volunteer immunized with JEV vaccine

BLAST searching result show that the L chain of mAb LZY3412 belongs to kappa (k) chain. We analyzed the nucleotide sequences of LZY3412 using the IMGT tool to identify their closest VH and Vk germline genes. The results show that LZY3412 originated from B-cell lineages. The LZY3412 VH gene was derived from IGHV4-39 and the Vk gene was from IGKV1D-39. We found that the encoding genes of LZY3412 closely resembled their corresponding germline gene segments. Notably, LZY3412 VH and Vk genes share 75.2 and 72.2% sequence identifies with the IGHV4-39*01 and IGKV1-39*01 germlines, respectively (Table 1). These results indicate that LZY3412 should be a somatically hypermutated mAb. The amino acid sequences of VH and VL of mAb LZY3412, as well as the CDR1, CDR2, and CDR3 amino acid sequences of VH and VL, are shown in Table 2.

**Table 1:** Genetic analysis of the heavy and light chain variable regions of mAb LZY3412.

	Variable regions	Variable region identity (%)	J region
V_H_	IGHV4-39	75.2	IGHJ6
V_L_	IGKV1D-39	72.2	IGKJ1

**Table 2:** The amino acid sequences of recombinant mAb LZY3412.

Chains	Domains	Sequences	Origination
H	CDR1	GGSISSSSY	This study
	CDR2	YYSGS	This study
	CDR3	CARGRIRLYYYYGMDVW	This study
L	CDR1	RASQSISSYLN	This study
	CDR2	AASSLQS	This study
	CDR3	CQQSYSTPGTF	This study
H	V	QLQLQESGPGLVKPSETLSLTCTVSGGSISSSSYYWGWIRQPPGKGLEWIGSIYYSGSTYYNPSLKSRVTISVDTSKNQFSLKLSSVTAADTAVYYCARGRIRLYYYYGMDVWGQGTTVTVSS	This study
L	V	DIQMTQSPSSLSASVGDRVTITCRASQSISSYLNWYQQKPGKAPKLLIYAASSLQSGVPSRFSGSGSGTDFTLTISSLQPEDFATYYCQQSYSTPGTFGQGTKVEIK	This study
H	C	ASTKGPSVFPLAPSSKSTSGGTAALGCLVKDYFPEPVTVSWNSGALTSGVHTFPAVLQSSGLYSLSSVVTVPSSSLGTQTYICNVNHKPSNTKVDKKVEPKSCDKTHTCPPCPAPELLGGPSVFLFPPKPKDTLMISRTPEVTCVVVDVSHEDPEVKFNWYVDGVEVHNAKTKPREEQYNSTYRVVSVLTVLHQDWLNGKEYKCKVSNKALPAPIEKTISKAKGQPREPQVYTLPPSRDELTKNQVSLTCLVKGFYPSDIAVEWESNGQPENNYKTTPPVLDSDGSFFLYSKLTVDKSRWQQGNVFSCSVMHEALHNHYTQKSLSLSPGK	AFR78282.1
L	C	RTVAAPSVFIFPPSDEQLKSGTASVVCLLNNFYPREAKVQWKVDNALQSGNSQESVTEQDSKDSTYSLSSTLTLSKADYEKHKVYACEVTHQGLSSPVTKSFNRGECS	AKL91149.1

### Recombinant mAb LZY3412 exhibits high-affinity with E proteins of JEV and ZIKV

**Figure 1:**
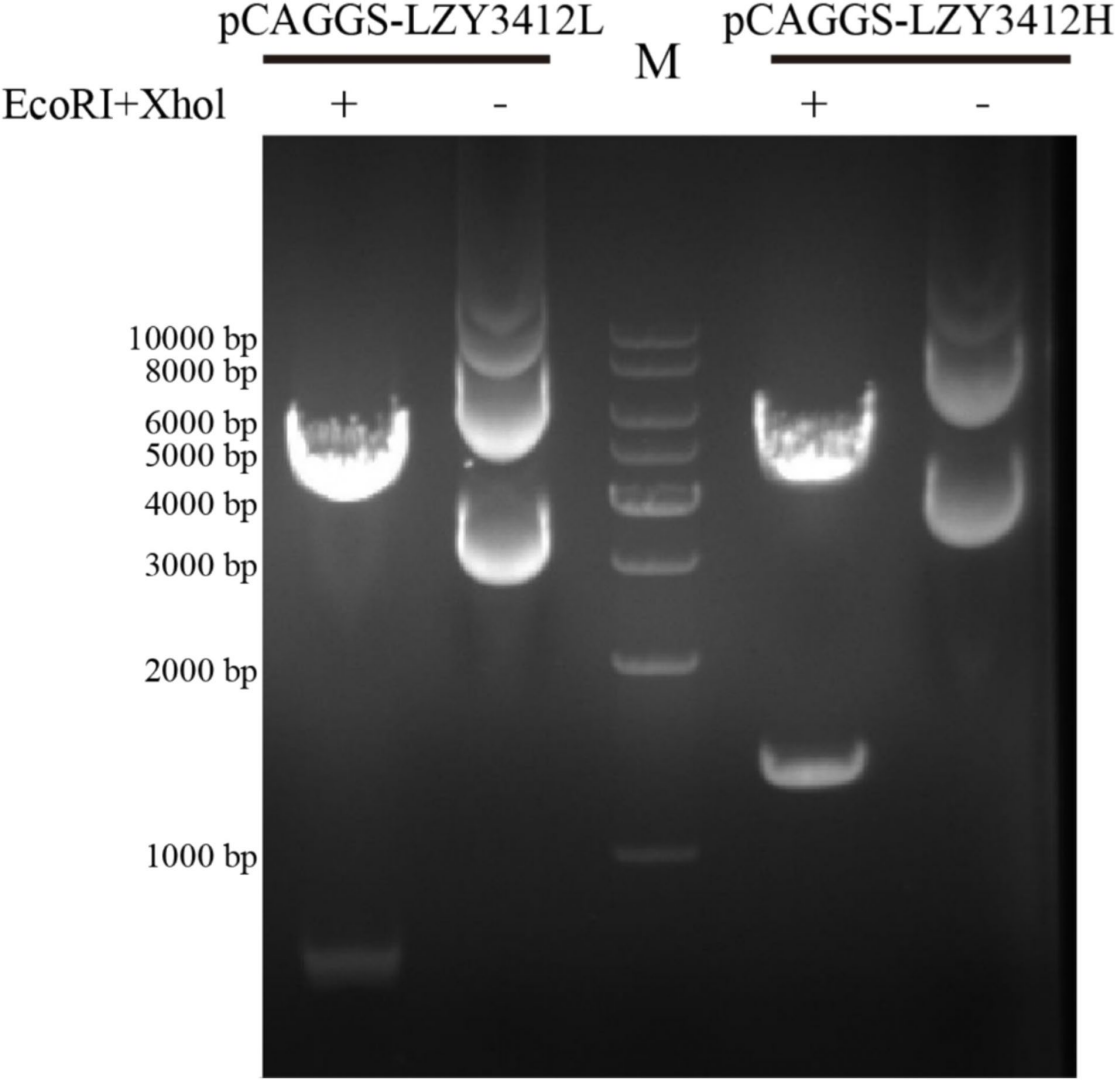
Agarose gel electrophoresis of two recombinant plasmids. The recombinant plasmids (pCAGGS-LZY3412L and pCAGGS-LZY3412H) were treated with two restriction endonucleases (*EcoR*I and *Xho*l) and then subjected to agarose gel electrophoresis. “M” indicates DNA marker.

**Figure 2:**
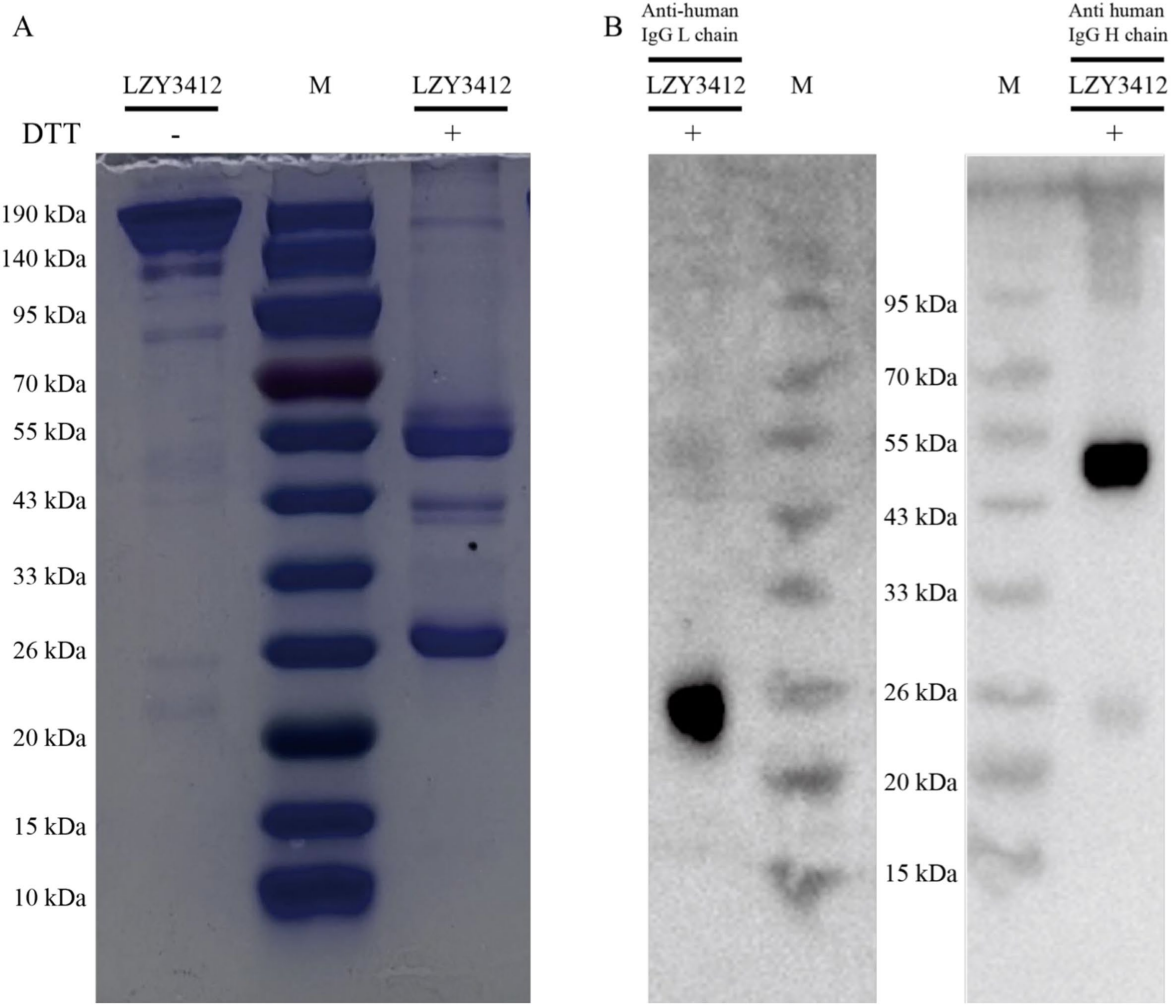
The SDS-PAGE and WB results of recombinant mAb LZY3412. **(A)** Recombinant mAb LZY3412 was subjected to SDS-PAGE electrophoresis at the absence or presence of DTT. **(B)** Recombinant mAb LZY3412 was subjected to SDS-PAGE electrophoresis at the presence of DTT, followed by WB. The light chain (k chain) and heavy chain of LZY3412 were detected using HRP-conjugated mAbs against the light chain and the heavy chain, respectively.

**Figure 3:**
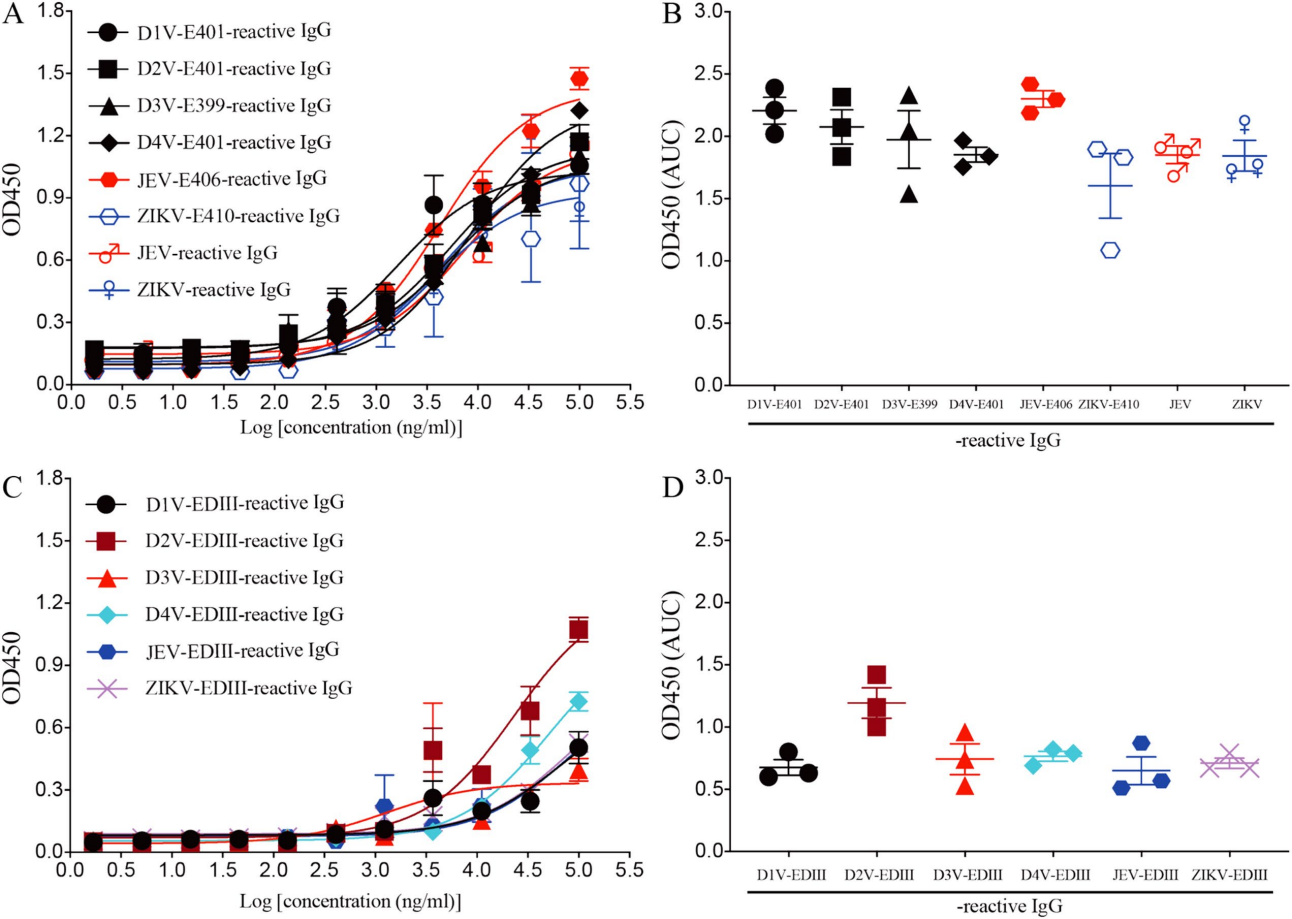
Recombinant mAb LZY3412 binds both JEV-E406 and ZIKV-E410 proteins. (**A–D**) 3-fold serially diluted LZY3412 (starting from 100 μg/mL) was added to D1V-E401-, D2V-E401-, D3V-E399-, D4V-E401-, JEV-E406-, ZIKV-E410-, JEV-, ZIKV-, D1V-EDIII-, D2V-EDIII-, D3V-EDIII-, D4V-EDIII-, JEV-EDIII-, or ZIKV-EDIII-coated ELISA plates as described in the section “Materials and Methods.” The levels of antigen-reactive IgG were measured using ELISA. Data are presented as the mean ± SEM. “AUC”: area under curve.

**Figure 4:**
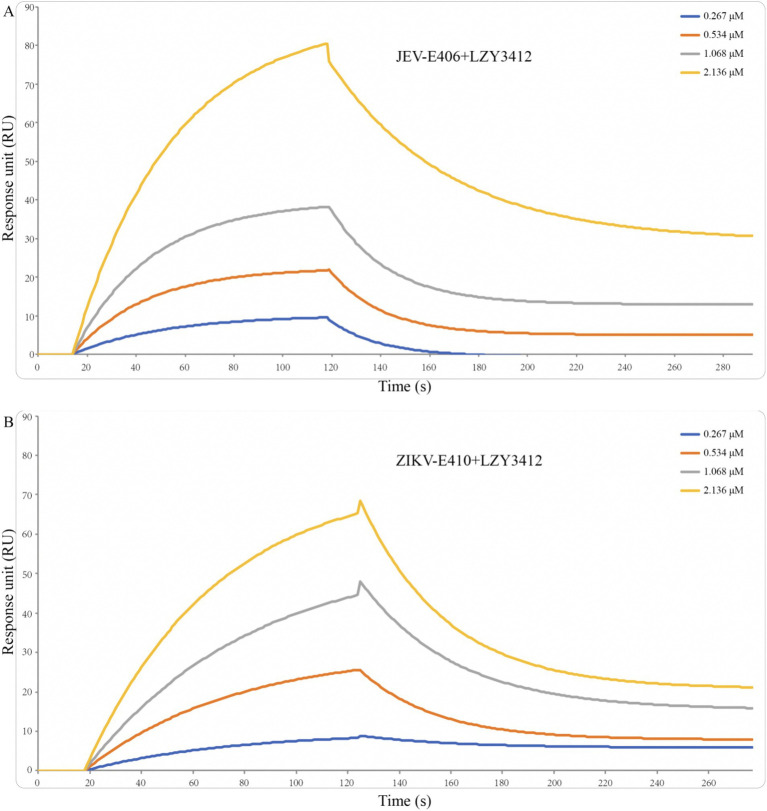
Binding affinity of recombinant mAb LZY3412 with JEV-E406 **(A)** and ZIKV-E410 **(B)** proteins. Recombinant JEV-E406 or ZIKV-E410 was coupled to LifeDisc^TM^ MetaSPR biosensors. Different concentrations (0.267, 0.534, 1.068, and 2.136 μM) of recombinant mAb LZY3412 flow through biosensor fixed with antigen. Association and dissociation profiles were fitted with WeSPR One software package assuming 1:1 binding model.

To investigate the function of this novel mAb LZY3412, we connected the VH and VL sequences of LZY3412 to the CH and CL sequences of two human IgGs, respectively, thus obtained the recombinant mAb LZY3412. The amino acid sequences of the CH and CL of two human IgGs are used for prepare recombinant mAb LZY3412 and shown in Table 2. The nucleotide sequences encoding the VH and CL of mAb LZY3412 were synthesized and cloned into recombinant pCAGGS plasmids containing genes encoding the CH and CL of two human IgGs, resulting in two recombinant plasmids pCAGGS-LZY3412H and pCAGGS-LZY3412L, respectively (Figure 1). The nucleotide sequences encoding the H and L chains of recombinant mAb LZY3412 are shown in Supplementary Table S2. After co-transfecting HEK-293 T cells with two recombinant plasmids, we detected H and L chains of recombinant mAb LZY3412 from the cell supernatant using SDS-PAGE and WB (Figure 2). We next evaluated the affinity of purified recombinant mAb LZY3412 with 6 recombinant viral proteins (JEV-E406, ZIKV-E410, D1V-E401, D2V-E401, D3V-E399, and D4V-E401) and live viral particles (JEV and ZIKV). The ELISA results show that recombinant mAb LZY3412 not only bind to JEV-E406 but also cross-recognize E proteins from JEV or DV, as the OD450 values are directly proportional to the concentration of recombinant mAb LZY3412 (Figure 3). As expected, recombinant mAb LZY3412 displayed high binding-affinity for JEV and ZIKV viral particles (Figure 3). Moreover, the binding ability of recombinant mAb LZY3412 to JEV-E406 protein is slightly higher than that of recombinant mAb LZY3412 to ZIKV-E410 protein. The surface plasmon resonance (SPR) results indicate that recombinant mAb LZY3412 has high-affinity with JEVE406 and ZIKV-E410 proteins, with a kinetically derived binding affinity (KD) values of 440 nM and 482.5 nM, respectively (Figure 4).

### Recombinant mAb LZY3412 efficiently neutralizes JEV and ZIKV infections *in vitro*

**Figure 5:**
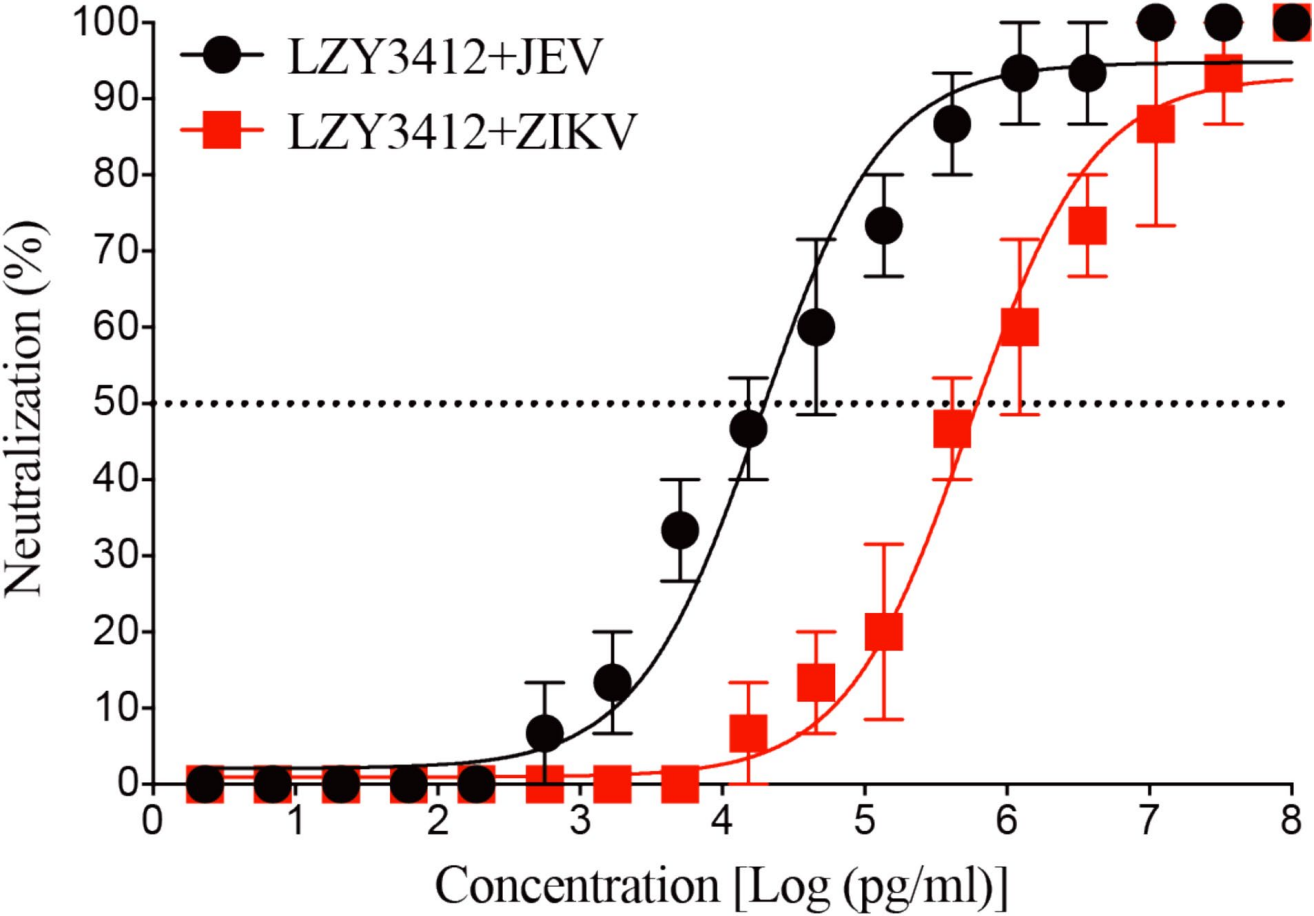
Recombinant mAb LZY3412 effectively neutralizes JEV and ZIKV infections *in vitro*. Recombinant mAb LZY3412 was tested for neutralization of JEV or ZIKV infections of Vero cells using PRNT as described in the Materials and Methods. Data are presented as the mean ± SEM.

Considering that recombinant mAb LZY3412 has high binding-affinity with the extracellular domain of the E proteins of JEV and ZIKV, we next to investigate whether recombinant mAb LZY3412 can block the entry of JEV/ZIKV into susceptible cells. In the present study, we evaluated the potential of recombinant mAb LZY3412 to prevent JEV and ZIKV infection *in vitro* using Vero cell-based PRNT. As a result, recombinant mAb LZY3412 neutralized both JEV and ZIKV infections in Vero cells in a concentration-dependent manner, indicating that recombinant mAb LZY3412 probably first blocked the binding of E protein to receptors on the cell surface, thereby stop the entry of JEV and ZIKV into the cells. The NT_50_ value of recombinant mAb LZY3412 for neutralizing JEV infection is 19.9 ng/mL, while the NT_50_ value for neutralizing ZIKV infection is 631 ng/ml (Figure 5).

### A single injection of recombinant mAb LZY3412 significantly increases the percent survival of mice infected with JEV or ZIKV

**Figure 6:**
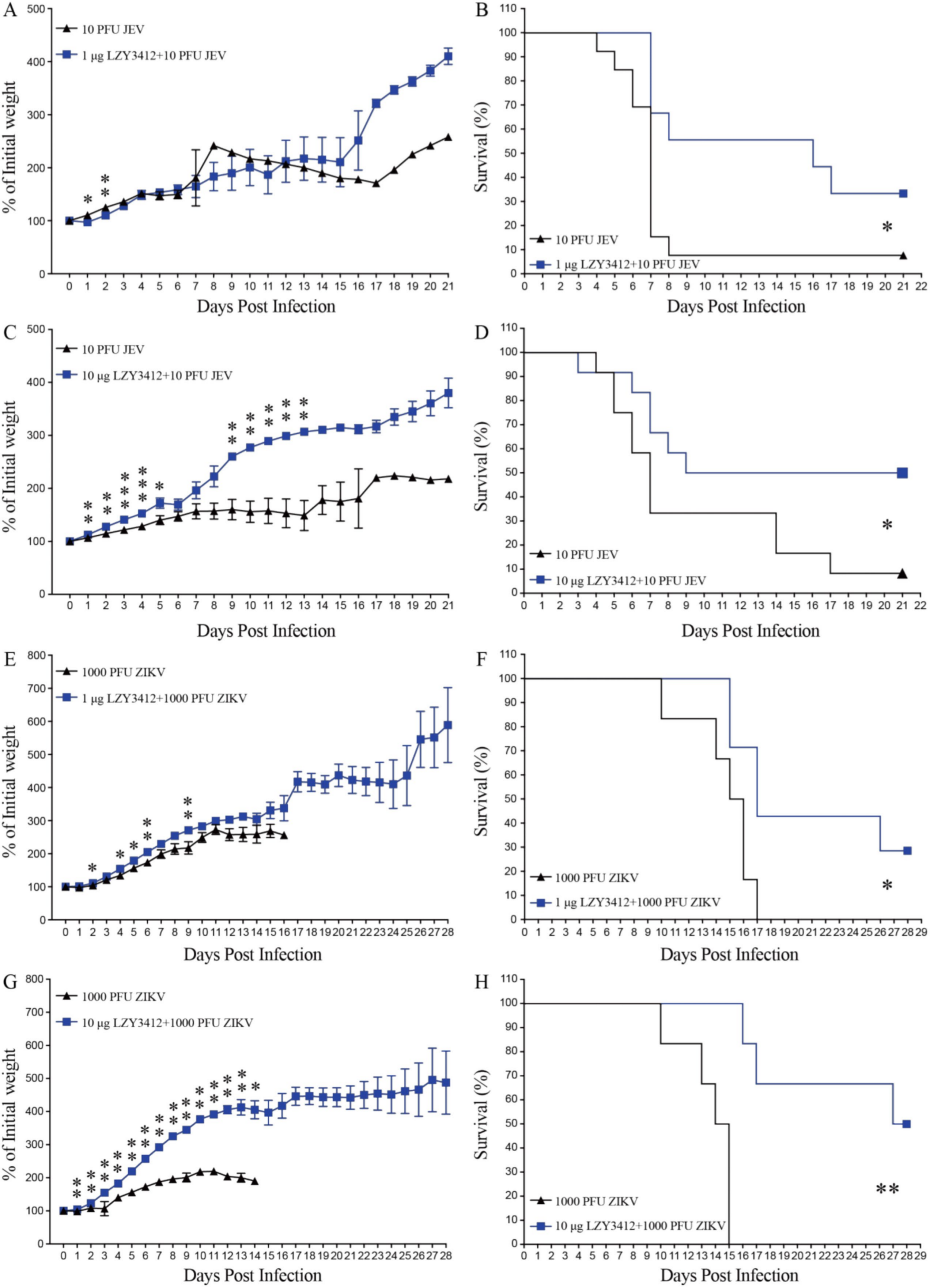
A single dose of mAb LZY3412 significantly increases the percent survival of neonatal mice infected with JEV or ZIKV. 1-day-old C57BL/6 naive mice were s.c. injected with 10 PFU of JEV or 1,000 PFU of ZIKV. Two hours later, mice were injected with 1 or 10 μg mAb LZY3412. Mouse weights and survival were recorded daily for 21 days or 28 days. Data are presented as the mean ± SEM. *p < 0.05, **p < 0.01, ***p < 0.001 by two-tailed Mann–Whitney *U* test **(A,C,E,G)** or log-rank test **(B,D,F,H)**.

**Figure 7:**
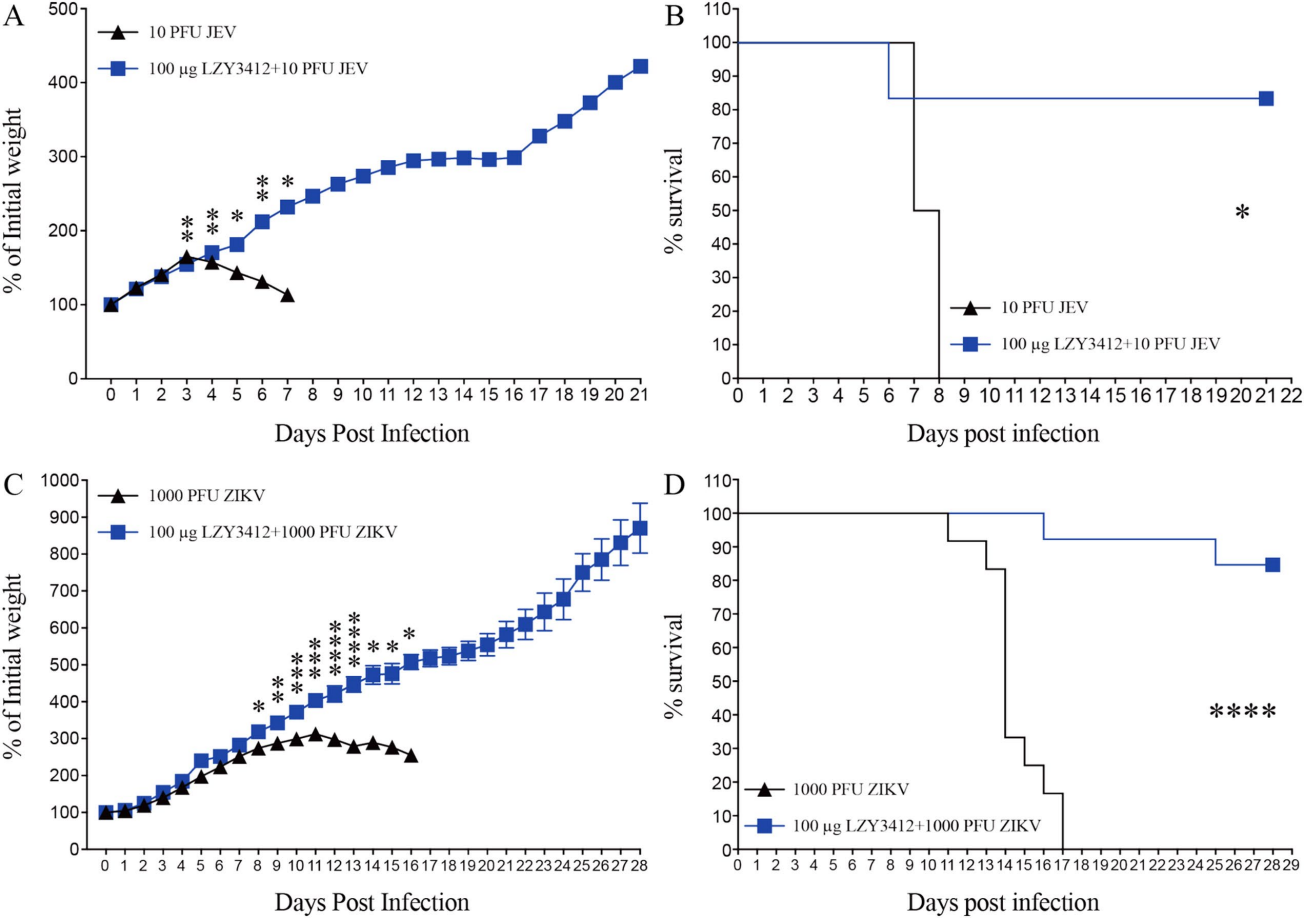
Hundred micrograms mAb LZY3412 protects most neonatal mice against lethal JEV or ZIKV challenge. 1-day-old C57BL/6 naive mice were s.c. injected with 10 PFU of JEV or 1,000 PFU of ZIKV. Two hours later, mice were injected with 100 μg LZY3412 mAb. Mouse weights and survival were recorded daily for 21 days or 28 days. Data are presented as the mean ± SEM. *p < 0.05, **p < 0.01, ***p < 0.001, ****p < 0.0001 by two-tailed Mann–Whitney *U* test **(A,C)** or log-rank test **(B,D)**.

We next determine whether application of recombinant mAb LZY3412 can protect mice against JEV or ZIKV challenge. Due to the resistance of adult WT mice to JEV and ZIKV infections whereas neonatal mice can support virus replication, which is attributable to the underdeveloped immune system. Particularly, 1-day-old neonatal mouse is an ideal model for evaluating the protective efficacy of Abs and antiviral agents against JEV/ZIKV because these neonatal mice can develop days-lasting viremia and even die couples of days post JEV or ZIKV attack. In the present study, we first infected 1-day-old neonatal mice with JEV or ZIKV, and then administered a single dose (1 or 10 μg) of purified recombinant mAb LZY3412 for survival study. As shown in Figure 6, most of the mice (>91%) in the control groups died from JEV infection, and more than half of the mice died within the first 2 weeks of viral challenge. In comparison, all the mice in control groups died from ZIKV infection within the first 17 days of viral challenge. As for JEV infection, the percentage survival rate of mice administered with 1 or 10 μg recombinant mAb LZY3412 were significantly higher than that of control mice (33.3% versus 7.7% survival, *p* = 0.0196; 50% versus 8.3% survival, *p* = 0.0444, respectively). Similarly, the percentage survival rate of ZIKV-infected mice given with 1 or 10 μg recombinant mAb LZY3412 were also significantly higher than that of control mice (28.6% versus 0, *p* = 0.0329; 50% versus 0, *p* = 0.0011) (Figure 6). In addition, application of recombinant mAb LZY3412 significantly attenuated the weight loss of JEV-infected and ZIKV-infected mice (Figure 6). We next tested whether giving more recombinant mAb LZY3412 could save the lives of more virus-infected mice. As expected, a single dose of 100 μg recombinant mAb LZY3412 increased the survival rates of mice infected with JEV or ZIKV to 83 and 84%, respectively (Figure 7). These data indicate that recombinant mAb LZY3412 mediates protection against both JEV and ZIKV infections *in vivo*.

### Application of recombinant mAb LZY3412 significantly reduce the serum viral loads of virus-infected neonatal mice

**Figure 8:**
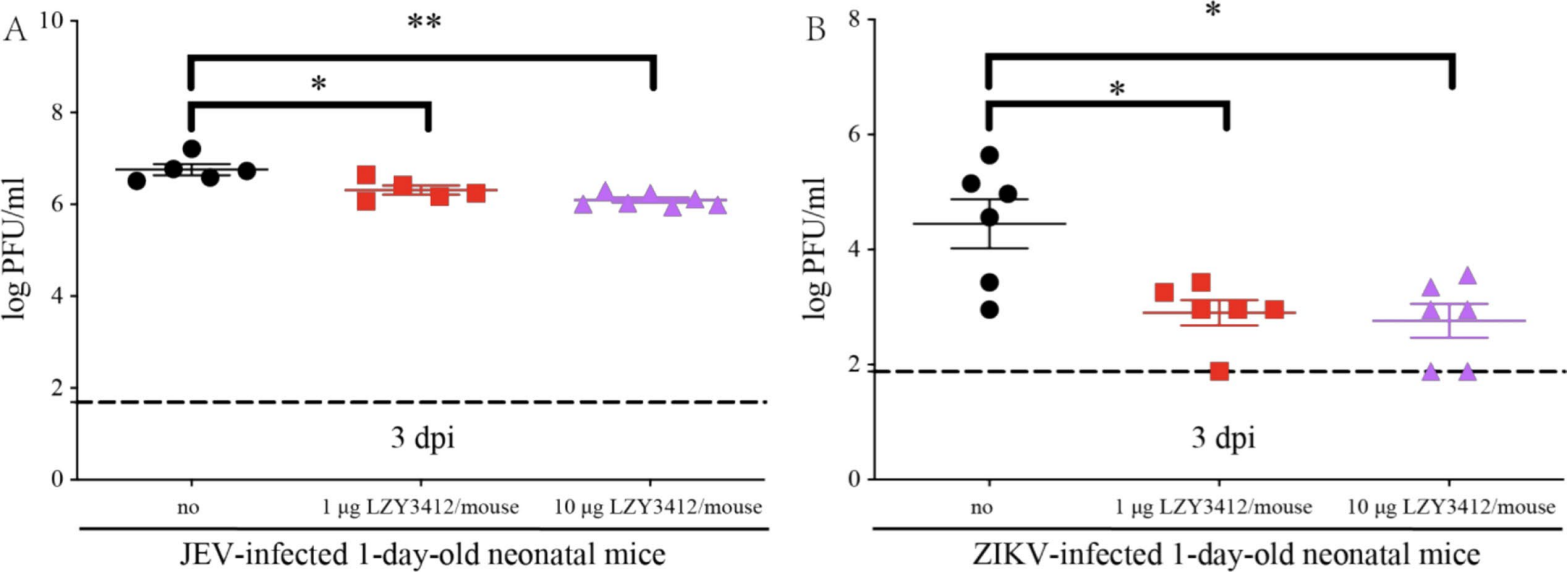
A single dose of mAb LZY3412 significantly reduces the tissue viral load of neonatal mice infected with JEV or ZIKV. 1-day-old C57BL/6 naive mice were s.c. injected with 10 PFU of JEV **(A)** or 1,000 PFU of ZIKV **(B)**. Two hours later, mice were injected with 1 or 10 μg mAb LZY3412. Three days post infection, the mice were sacrificed and the mouse sera were collected for detecting viral load using PFA. Data are presented as the mean ± SEM. *p < 0.05, **p < 0.01 by two-tailed Mann–Whitney *U* test.

We further explore whether the protective role of recombinant mAb LZY3412 in mice is associated with the reduction of tissue viral burden. We injected 1 or 10 μg of recombinant mAb LZY3412 into 1-day-old neonatal mice infected with JEV or ZIKV and detected the serum viral loads 3 days post virus infection. The serum viral loads of JEV-infected mice receiving 1 or 10 μg recombinant mAb LZY3412 were significantly lower than that of control mice (2.041 × 10^6^ versus 5.754 × 10^6^ PFU/ml, *p* = 0.0317; 0.977 × 10^6^ versus 5.754 × 10^6^ PFU/ml, *p* = 0.0025, respectively). Similarly, the serum viral loads of ZIKV-infected mice given with 1 or 10 μg recombinant mAb LZY3412 were also significantly lower than that of control mice (0.794 × 10^3^ versus 2.818 × 10^4^ PFU/ml, *p* = 0.0281; 0.575 × 10^3^ versus 2.818 × 10^4^ PFU/ml, *p* = 0.0238) (Figure 8). Thus, these data indicate that recombinant mAb LZY3412 plays the protective activity against both JEV and ZIKV infections via restricting virus replication *in vivo*.

### Epitope mapping of LZY3412 by molecular docking

**Figure 9:**
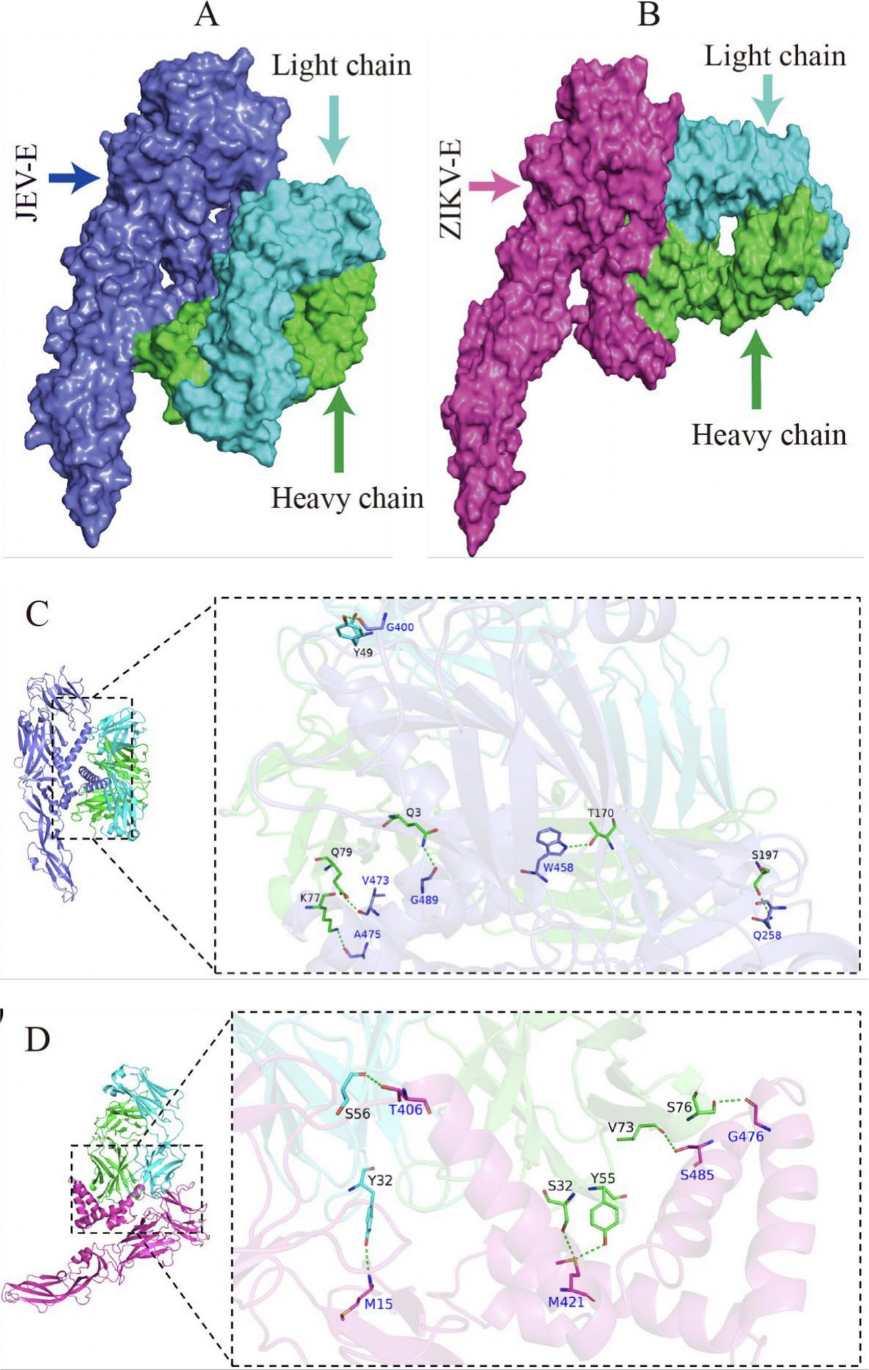
Antigen–antibody docking complex and spatial docking sites. **(A)** Structure of JEV-E and LZY3412 docking complex. **(B)** Structure of ZIKV-E and LZY3412 docking complex. **(C)** The 3D interaction mode between JEV-E and LZY3412. **(D)** The 3D interaction mode between ZIKV-E and LZY3412.

**Figure 10:**
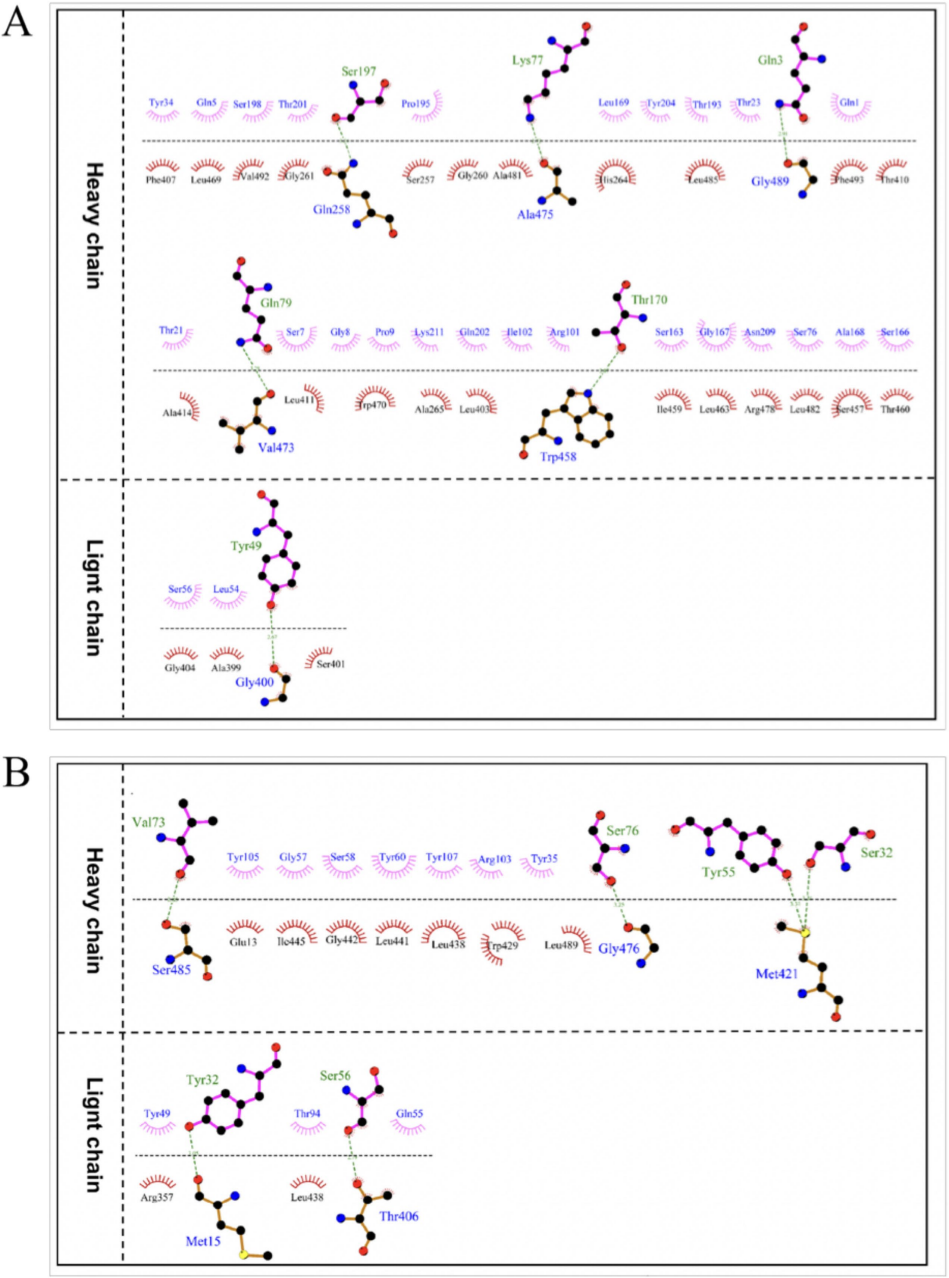
The interaction mode between antigen and antibody binding regions. **(A)** The 2D interaction mode between JEV-E protein and LZY3412 mAb. **(B)** The 2D interaction mode between ZIKV-E protein and LZY3412 mAb. Toothed amino acids exhibit hydrophobic interactions, green dashed lines represent hydrogen bonding interactions, and red dashed lines represent salt bridge interactions.

The optimal docking score for JEV-E and recombinant mAb LZY3412 molecule is −299.80, with a Confidence Score of 0.952. This result indicates that the complex model obtained by docking has high credibility, and the optimal model for protein molecule docking is shown in Figure 9A. Subsequently, the interaction patterns of spatial structure binding sites and binding regions between JEV-E protein and recombinant mAb LZY3412 molecule was further analyzed. Figures 9C, 10A show the interaction patterns between JEV-E protein and recombinant mAb LZY3412. It can be seen from the figures that JEV-E protein forms 5 sets of hydrogen bonds with the H chain of recombinant mAb LZY3412 and 1 set of hydrogen bonds with the L chain. Among the specific hydrogen bonds formed by amino acids between two proteins, Gln^258^, Gly^400^, Trp^458^, Val^473^, Ala^475^, and Gly^489^ are from JEV-E protein; the amino acid residues from recombinant mAb LZY3412 H chain include Gln^3^, Lys^77^, Gln^79^, Thr^170^, and Ser^197^; the amino acid in the L chain is Tyr^49^ (Figure 10A). In addition, there are numerous hydrophobic interactions between the two proteins that promote further stable binding, forming an Ab-antigen complex (Figure 10A). It is worth noting that the hydrogen bond formed by the amino acid residue Gly^400^ from JEV-E protein (present in the EDIII region) and the amino acid residue Tyr^49^ from the CDR2 of the L chain from mAb LZY3412, as well as the hydrophobic bond formed by the amino acid residue Ala^399^ from JEV-E protein (present in the EDIII region) and the amino acid residue Leu^54^ from the CDR2 of the L chain from mAb LZY3412, may play a key role in mAb LZY3412 specific blocking of JEV infection.

The optimal docking result between ZIKV-E protein and recombinant mAb LZY3412 has a Docking Score of −297.38 and a Confidence Score of 0.950. This result indicates that the complex model obtained by docking has high credibility, and the optimal model result for protein molecule docking is shown in Figure 9B. Subsequently, the interaction patterns of the spatial structure binding sites and binding regions between ZIKV-E protein and recombinant mAb LZY3412 was further analyzed. Figures 9D, 10B show the interaction patterns between ZIKV-E and recombinant mAb LZY3412. It can be seen from the figures that ZIKV-E protein forms four sets of hydrogen bonds with the H chain of recombinant mAb LZY3412 and two sets of hydrogen bonds with the L chain. Among the specific hydrogen bonds formed by amino acids between two proteins, those from ZIKV-E protein include Met^15^, Thr^406^, Met^421^, Gly^476^, and Ser^485^; The amino acid residues from the H chain of recombinant mAb LZY3412 include Ser^32^, Tyr^55^, Val^73^, and Ser^76^; the amino acids in the L chain include Tyr^32^ and Ser^56^ (Figure 10B). In addition, there are numerous hydrophobic interactions between the two proteins that promote further stable binding, forming an antibody–antigen complex (Figure 10B). The hydrophobic bond formed by the amino acid residue Arg^357^ from ZIKV-E protein (present in the EDIII region) and the amino acid residue Tyr^49^ from the CDR2 of the L chain from mAb LZY3412 may play a key role in mAb LZY3412 specific blockade of ZIKV infection.

### Two amino acid substitutions reduce the binding capacity of recombinant mAb LZY3412 to JEV-E protein

**Figure 11:**
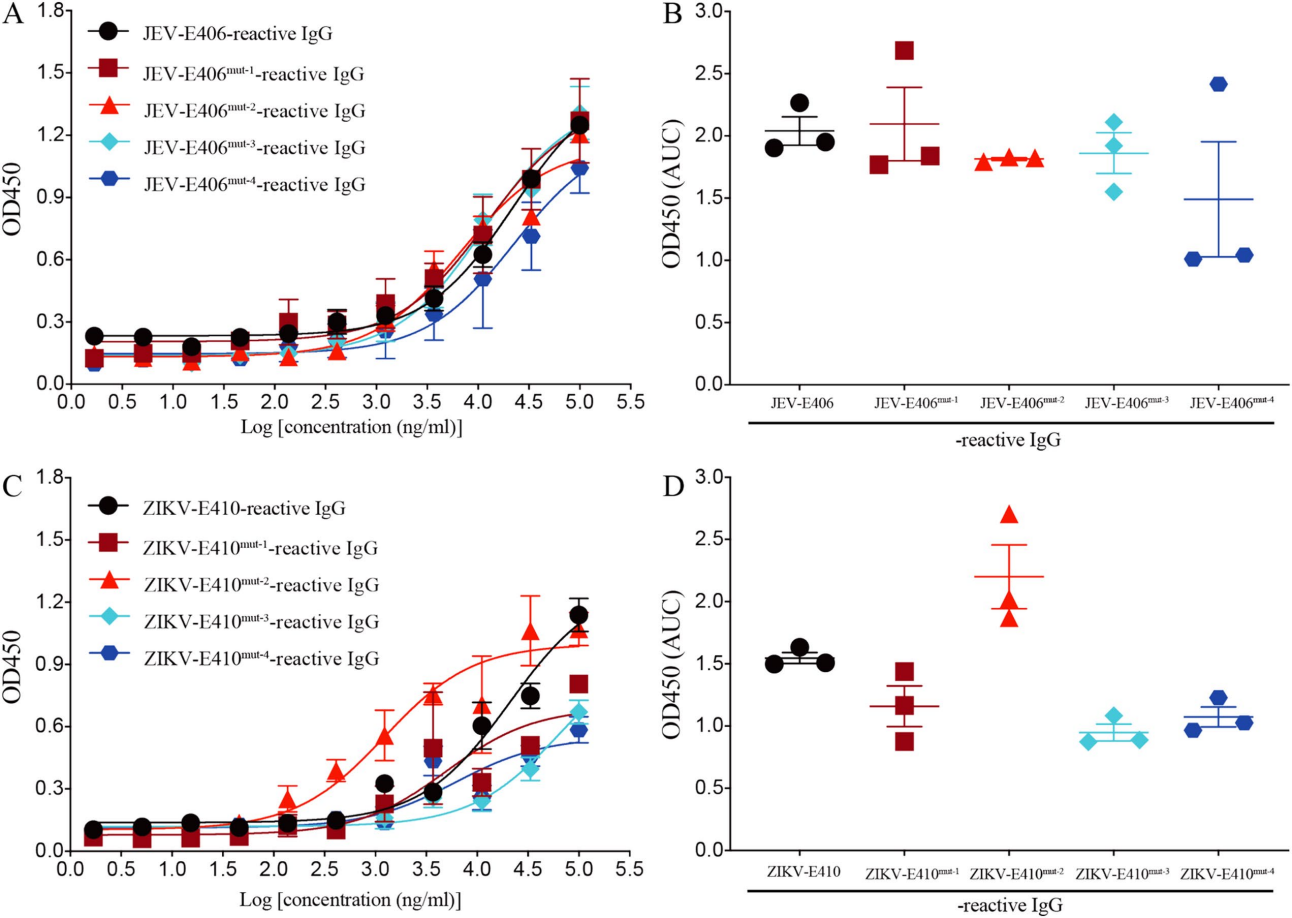
Recombinant mAb LZY3412 binds to the mutants of JEV-E406 and ZIKV-E410 proteins. **(A–D)** 3-fold serially diluted LZY3412 (starting from 100 μg/mL) was added to JEV-E406-, JEV-E406^mut-1^-, JEV-E406^mut-2^-, JEV-E406^mut-3^-, JEV-E406^mut-4^-, ZIKV-E410-, ZIKV-E410^mut-1^-, ZIKV-E410^mut-2^-, ZIKV-E410^mut-3^-, or ZIKV-E410^mut-4^-coated ELISA plates as described in the Materials and Methods. The levels of antigen-reactive IgG were measured using ELISA. Data are presented as the mean ± SEM. “AUC”, area under curve.

Based on the molecular docking results, three amino acids (Gln^258^, Ala^399^, and Gly^400^) in JEV-E406 protein may play the important role in the recognition by recombinant mAb LZY3412. To explore whether these amino acids are truly involved in the epitope formation, we replaced these amino acids individually or simultaneously with Ala or Arg, thus constructing four JEV-E406 mutants. The ELISA results showed that Gln^258^Ala substitution had no significant effect on the binding of LZY3412 to JEV-E406 protein mutant, while Ala^399^Arg or/and Gly^400^Ala substitution could reduce the recognition of JEV-E406 protein mutant by LZY3412, suggesting that Ala^399^ and Gly^400^ should be key amino acid sites for LZY3412 to recognize JEV-E406 protein (Figures 11A,B).

### Two amino acid substitutions reduce the binding capacity of recombinant mAb LZY3412 to ZIKV-E protein

The above molecular docking results show that three amino acids (Met^15^, Arg^357^, and Thr^406^) in ZIKV-E410 protein are potential recognition sites by mAb LZY3412. To investigate whether these amino acids are key determinants in the epitope recognized by LZY3412, we substituted these amino acids individually or simultaneously with Ala or Arg, thus obtaining four ZIKV-E410 protein mutants. As shown in Figures 11C,D, Arg^357^Ala substitution did not reduce the binding affinity of LZY3412 to ZIKV-E410 protein mutant, whereas Met^15^Ala or/and Thr^406^Ala substitutions reduced the binding affinity of LZY3412 to ZIKV-E410 protein mutants, suggesting that Met^15^ and Thr^406^ should be key amino acid sites for LZY3412 to recognize ZIKV-E410 protein.

## Discussion

The inactivated and live-attenuated JEV vaccines have been approved for use for decades, but there is currently no approved ZIKV vaccine available. Recent studies demonstrated that JEV vaccination or JEV infection-induced Abs can enhance ZIKV infection *in vitro* and *in vivo*, and vice versa, i.e., ADE of infection effects (Chen et al., 2020; Wang et al., 2020), which bring many difficulties to develop an effective and safety vaccines against JEV and ZIKV. Therefore, it is urgent to develop the effective antiviral agents or Ab drugs. In the present study, we obtained a novel mAb LZY3412 by sequencing the B cells of JEV vaccine-vaccinated volunteer. Our *in vitro* and *in vivo* experimental results demonstrate that mAb LZY3412 belongs to a broad-spectrum neutralizing mAb, as it can bind to the E proteins of DV, JEV and ZIKV, and block both JEV and ZIKV infections *in vitro* with good neutralizing activity. Most importantly, the mouse challenge experiment results show that mAb LZY3412 has preventive/therapeutic effects on JEV and ZIKV infections.

Given the co-circulation of many flaviviruses in some tropical/subtropical countries/territories, it is particularly important to develop broad-spectrum human mAbs that have cross-neutralizing effects on multiple flaviviruses. So far, a growing number of human mAbs that have neutralizing activity against both ZIKV and DENV have been identified (Robbiani et al., 2017; Yu et al., 2017; Niu et al., 2019; Dussupt et al., 2020; Zhang et al., 2020; Zhao et al., 2020), however, the numbers of fully human JEV-neutralizing mAb is very rare (Fernandez et al., 2018; Ozawa et al., 2018), and there are no reports of human mAbs that have neutralizing effects on both ZIKV and JEV. In the present study, we first isolated a human B cell induced by JEV vaccine inoculation using ZIKV antigen (E410) and then obtained the V region sequences of mAb LZY3412 through BCR gene sequencing. To evaluate the neutralization effects on JEV and ZIKV of LZY3412, we used a standard PRNT and 1-day-old neonatal mouse model to measure JEV/ZIKV infection and neutralization *in vitro* and *in vivo*, respectively. Although the NT_50_ value of LZY3412 against JEV (19.9 ng/mL) was slightly lower than the NT_50_ values of three mouse-derived JEV-neutralizing mAbs [17BD3-2 (4.9 ng/mL), 2H4 (1.2 ng/mL), and 2F2 (1.4 ng/mL)] (Calvert et al., 2019; Qiu et al., 2018), the NT_50_ value of mAb LZY3412 against JEV is superior to the NT_50_ values of five human-derived JEV-neutralizing mAbs reported in two recent studies (Fernandez et al., 2018; Ozawa et al., 2018). In one study, the researchers established three fully human WNV-neutralizing mAbs from the peripheral blood mononuclear cells of inactivated JEV vaccine-inoculated individuals, among them, mAb WN_83 has an NT_50_ of 4.9 μg/mL for WNV and 13.1 μg/mL for JEV (Ozawa et al., 2018). Another study reported four human mAbs (hJEV-11, hJEV-69, hJEV-75, and hJEV-80). Among them, mAb hJEV-75 has the best NT_50_ value (228 ng/mL) for JEV (Fernandez et al., 2018). Consistent with a previous study showing that pre-administration of 10 μg of hJEV-69 or hJEV-75 to 5-week-old C57BL/6 mice can provide partial protective effects during JEV challenge (Fernandez et al., 2018), application of mAb LZY3412 to 1-day-old C57BL/6 mice exhibited the therapeutic potential for JEV and ZIKV infections. Specifically, a single injection of 1 μg mAb LZY3412 reduced the viral titer in the serum of JEV-infected 1-day-old mice by around 2 folds at 3 days after virus challenge, while a single injection of 10 μg LZY3412 reduced the viral titer in serum by approximately 5 folds (Figure 8A). Correspondingly, in ZIKV-infected mouse model, a single injection of 1 μg mAb LZY3412 reduced the serum viral load by 34 folds, while 10 μg mAb LZY3412 reduced the serum viral load by 48 folds (Figure 8B).

E protein is the primary target of neutralizing Abs and is composed of three ectodomains: EDI, which links EDII and EDIII together (Modis et al., 2004; Dai et al., 2016). We next analyzed mAb LZY3412 epitope on E proteins of JEV/ZIKV by using computational structural modeling and constructing E protein mutants. The critical residues (Ala^399^ and Gly^400^) recognized by mAb LZY3412 exist in EDIII of JEV-E protein, and are not overlapped with the epitopes previously explored. However, two critical amino acid residues (Met^15^ and Thr^406^) where LZY3412 recognizes ZIKV-E protein are not located in the EDIII, but in EDI and other region of E protein, respectively. In addition, although molecular docking analysis suggest that the amino acid residue Arg^357^ from ZIKV-E protein (present in the EDIII region) can form the hydrophobic bond with the amino acid residue Tyr^49^ from the CDR2 of the L chain from mAb LZY3412, incorporation of Ala to this site did not weaken the binding affinity of LZY3412 to ZIKV-E410 protein mutant. In fact, the majority of previously reported flavivirus E protein-specific neutralizing mAbs are EDIII-targeted (Stettler et al., 2016; Sapparapu et al., 2016; Magnani et al., 2017; Robbiani et al., 2017; Wang et al., 2017; Keeffe et al., 2018; Niu et al., 2019; Dussupt et al., 2020; Zhao et al., 2020), whereas only a few neutralizing mAbs are EDII-targeted (Dejnirattisai et al., 2015; Deng et al., 2011). For instance, human mAb Z004 recognize Glu^393^ and Lys^394^ (Robbiani et al., 2017) whereas human mAb Z021 recognizes the EDI-EDIII hinge region, the BC loop, the DE loop, and the FG loop of ZIKV-EDIII (Keeffe et al., 2018). Human mAb ZKA190 epitope consists of the LR of EDIII (the BC, DE and FG loops), as well as part of the EDI-EDIII linker (Stettler et al., 2016; Wang et al., 2017). Human mAb 7B3 recognizes residues Thr^335^, Gly^337^, Glu^370^, and Asn^371^ as well (Niu et al., 2019). In addition, MZ4 targets a novel site of vulnerability centered on the EDI-EDIII linker region of ZIKV (Dussupt et al., 2020). One study revealed two classes of broadly neutralizing Abs which target the conserved fusion loop epitope (FLE) in DII or protein E dimer epitope (EDE) in DI/II of flaviviruses (Fibriansah and Lok, 2016). The present data suggest that mAb LZY3412 probably recognizes JEV and ZIKV with different amino acid residue positions in E protein (Met^15^ for ZIKV-EDI, Ala^399^ and Gly^400^ for JEV-EDIII, respectively). This is not surprising, because Robbiani et al. (2017) had reported a human mAb Z004 which recognizes Glu^393^ and Lys^394^ residues of ZIKV-EDIII whereas contacts Glu^384^ and Lys^385^ residues of DENV-EDIII. We also found that recombinant mAb LZY3412 can bind to the extracellular domain of the E proteins and EDIIIs of four serotypes of DV, and its binding-affinity to the former is stronger than that to the latter, suggesting that this mAb may also recognize the amino acid residues located in other regions of the E protein. To be honest, due to the limitations of the present experimental methods, we did no identify all the amino acid residues that this mAb can bind to, as well as the mechanism by which this mAb neutralizes JEV and ZIKV infections.

In conclusion, mAb LZY3412 has neutralizing effects on multiple flaviviruses (at least JEV and ZIKV) and may belong to pan-flavivirus broad-spectrum neutralizing mAb. There are two potential applications from the present findings: (1) mAb LZY3412 is a broad-spectrum neutralizing mAb which could be further developed in preclinical and clinical settings; (2) the potential epitope recognized by mAb LZY3412 could guide the development of effective vaccine candidate capable of inducing mAb LZY3412-like Ab response. We therefore expect that mAb LZY3412 has a druggability and could be developed as a therapeutic candidate in the future.

## Data Availability

The original contributions presented in the present study are included in the article/Supplementary material, further inquiries can be directed to the corresponding author.
