# Supplementary material for: A human monoclonal antibody isolated from Japanese encephalitis virus vaccine-vaccinated volunteer neutralizing various flaviviruses
Source: Front Microbiol. 2024 Dec 23;15:1508923. doi: 10.3389/fmicb.2024.1508923 (PMC11701783; doi:10.3389/fmicb.2024.1508923)
Supplement: Supplementary file 1 [file Data_Sheet_1.pdf]

A human monoclonal antibody isolated from Japanese encephalitis virus vaccine-vaccinated volunteer neutralizing various flaviviruses

Dong Chen[1, 2, 3 †], Jiayi Zhang[1, 2, 3 †], Yusha Liu[1 †], Jiayang Zhu[1†], Jie Chen[1], Hongxia Ni[4], Jinsheng Wen[1]*

[1] School of Basic Medical Sciences, Health Science Center, Ningbo University, Ningbo, China

[2] Wenzhou Central Blood Station, Wenzhou, China

[3] Key Laboratory of Laboratory Medicine, Ministry of Education, Zhejiang Provincial Key Laboratory of Medical Genetics, College of Laboratory Medicine and Life sciences, Wenzhou Medical University, Wenzhou, China

[4] Ningbo Municipal Center for Disease Control and Prevention, Ningbo, China

[†] These authors contributed equally to this work.

* CORRESPONDENCE. Jinsheng Wen, wenjinsheng@nbu.edu.cn

Supplementary materials

TABLE S1. The amino acid sequence alignment of the E protein ectodomains of six flaviviruses

Proteins	Amino acid sequences
D1V-E401	MRCVGIGNRDFVEGLSGATWVDVVLEHGSCVTTMAKDKPTLDIELLKTEVTKPAVLRKLC<br>IEAKISNTTTDSRCPTQGEATLVEEQDANFVCRRTFVDRGWGNGCGLFGKGSLITCAKFKC<br>VTKLEGKIVQYENLKYSVIVTVHTGDQHQVGNETTEHGTIATITPQAPTSEIQLTDYGALTL<br>DCSPRTGLDFNEMVLLTMKEKSWLVHKQWFLDLPLPWTSGASTPQETWNREDLLVTFKT<br>AHAKKQEVVVLGSQEGAMHTALTGATEIQTSGTTKIFAGHLKCRLKMNKLTLKGMSYVM<br>CTGSFKLEKEVAETQHGTVLVQVKYEGTDAPCKIPFSTQDEKGVTQNGRLITANPIVTDKE<br>KPVNIEAEPPFGESYIVVGAGEKALKLSWFKKGSSIGKM
D2V-E401	MRCIGISNRDFVEGVSGGSWVDIVLEHGSCVTTMAKNKPTLDFELIKTEAKQPATLRKYCIE<br>AKLTNTTTDSRCPTQGEPSLNEEQDKRFVCKHSMVDRGWGNGCGLFGKGGIVTCAMFTC<br>KKNMKGKVVQPENLEYTIVITPHSGEEHAVGNDTGKHGKEIKITPQSSITEAELTGYGTVT<br>MECSPRTGLDFNEMVLLQMENKAWLVHRQWFLDLPLPWLPGADTQGSNWIQKETLVTFK<br>NPHAKKQDVVVLGSQEGAMHTALTGATEIQMSSGNLLFTGHLKCRLRMDKLQLKGMSYS<br>MCTGKFKVVKEIAETQHGTIVIRVQYEGDGSPCKIPFEIMDLEKRHVLGRLITVNPIVTEKDS<br>PVNIEAEPPFGDSYIIIGVEPGQLKLNWFKKGSSIGQM
D3V-E399	MRCVGVGNRDFVEGLSGATWVDVVLEHGGCVTTMAKNKPTLDIELQKTEATQLATLRKL<br>CIEGKITNITTDSRCPTQGEAILPEEQDQNYVCKHTYVDRGWGNGCGLFGKGSLVTCAKFQ<br>CLESIEGKVVQHENLKYTVIITVHTGDQHQVGNETQGVTAEITSQASTAEAILPEYGTLGLE<br>CSPRTGLDFNEMILLTMKNKAWMVHRQWFFDLPLPWTSGATTKTPTWNRKELLVTFKNA<br>HAKKQEVVVLGSQEGAMHTALTGATEIQTSGGTSIFAGHLKCRLKMDKLKLKGMSYAMC<br>LNTFVLKKEVSETQHGTILIKVEYKGEDAPCKIPFSTEDGQGKAHNGRLITANPVVTKKEEP<br>VNIEAEPPFGESNIVIGIGDKALKINWYRKGSSIGKM
D4V-E401	MRCVGVGNRDFVEGVSGGAWVDLVLEHGGCVTTMAQGKPTLDFELIKTTAKEVALLRTY<br>CIEASISNITTATRCPTQGEPYLKEEQDQQYICRRDVVDRGWGNGCGLFGKGGVVTCAKFS<br>CSGKITGNLVQIENLEYTVVVTVHNGDTHAVGNDIPNHGVTATITPRSPSVEVKLPDYGELT<br>LDCEPRSGIDFNEMILMKMKKKTWLVHKQWFLDLPLPWAAGADTSEVHWNYKERMVTF<br>KVPHAKRQDVTVLGSQEGAMHSALTGATEVDSGDGNHMFAGHLKCKVRMEKLRIKGMS<br>YTMCSGKFSIDKEMAETQHGTTVVKVKYEGAGAPCKVPIEIRDVNKEKVVGRIISSTPFAE<br>YTNSVTNIELEPPFGDSYIVIGVGDSALTLHWFRKGSSIGKM
JEV-E406	FNCLGMGNRDFIEGASGATWVDLVLEGDSCLTIMANDKPTLDVRMINIEASQLAEVRSYC<br>YHASVTDISTVARCPTTGEAHNEKRADSSYVCKQGFTDRGWGNGCGFFGKGSIDTCAKFS<br>CTSKAIGRTIQPENIKYKVGIFVHGTTTSENHGNYSAQVGASQAAKFTVTPNAPSVALKLG<br>DYGEVTLDCEPRSGLNTEAFYVMTVGSKSFLVHREWFHDLALPWTSPSSTAWRNRELLME<br>FEGAHATKQSVVALGSQEGGLHHALAGAIVVEYSSSVMLTSGHLKCRLKMDKLALKGTT<br>YGMCTEKFSFAKNPVDTGHGTVVIELSYSGSDGPCKIPIVSVASLNDMTPVGRLVTVNPFV<br>ATSSANSKVLVEMEPPFGDSYIVVGRGDKQINHHWHKAGSTLGKA
ZIKV-E410	IRCIGVSNRDFVEGMSGGTWVDVVLEHGGCVTVMAQDKPTVDIELVTTTVSNMAEVRSY<br>CYEASISDMASDSRCPTQGEAYLDKQSDTQYVCKRTLVDRGWGNGCGLFGKGSLVTCAK<br>FACSKKMTGKSIQPENLEYRIMLSVHGSQHSGMIVNDTGHETDENRAKVEITPNSPRAEAT<br>LGGFGSLGLDCEPRTGLDFSDLYYLTMNNKHWLVHKEWFHDIPLPWHAGADTGTPHWNN<br>KEALVEFKDAHAKRQTVVVLGSQEGAVHTALAGALEAEMDGAKGRLSSGHLKCRLKMD<br>KLRLKGVSYSLCTAAFTFTKIPAETLHGTVTVEVQYAGTDGPCKVPAQMAVDMQTLTPVG<br>RLITANPVITESTENSKMMLELDPPFGDSYIVIGVGEKKITHHWHRSGSTIGKA

TABLE S2. DNA sequence encoding the H chain or L chain of mAb LZY3412

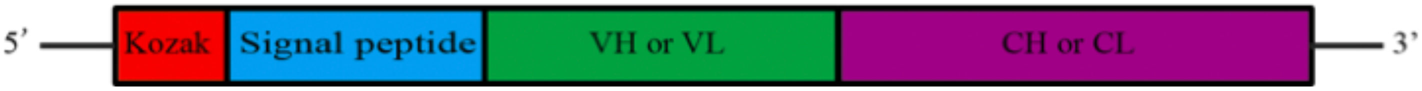

LZY3412H

5'-
gccaccATGAAGCACCTGTGGTTCTTCCTGCTCCTGGTGGCCGCCCCTAGATGGGTCCTGTCCCA
ACTGCAGCTGCAAGAGAGCGGCCCCGGCCTGGTGAAGCCTAGCGAGACCCTGAGCCTGACC
TGCACCGTGAGCGGCGGCAGCATCAGCAGCAGCAGCTACTACTGGGGCTGGATCAGACAGC
CCCCCGGCAAGGGCCTGGAGTGGATCGGCAGCATCTACTACAGCGGCAGCACCTACTACAA
CCCTAGCCTGAAGAGCAGAGTGACCATCAGCGTGGACACAAGCAAGAATCAGTTCAGCCTG
AAGCTGAGCAGCGTGACCGCCGCCGACACCGCCGTGTACTACTGCGCTAGAGGCAGAATCA
GACTGTACTATTACTACGGCATGGACGTGTGGGGCCAAGGCACCACCGTGACCGTGAGCAG
CGCTAGCACCAAGGGCCCTAGCGTCTTTCCCCTCGCCCCTAGCAGCAAGAGCACAAGCGGCG
GCACAGCCGCCCTGGGCTGCCTGGTCAAGGACTATTTCCCTGAGCCCGTGACCGTGTCCTGG
AACAGCGGCGCCCTCACAAGCGGCGTGCACACCTTCCCCGCCGTGCTGCAGAGCAGCGGCC
TGTACAGCCTGAGCAGCGTGGTGACCGTGCCTAGCAGCAGCCTGGGCACACAGACCTACAT
CTGCAACGTGAACCACAAGCCTAGCAACACCAAGGTGGACAAGAAGGTGGAGCCCAAAAG
CTGCGACAAGACCCACACCTGCCCTCCCTGCCCTGCCCCCGAGCTGCTGGGCGGCCCTAGCG
TGTTTCTGTTCCCCCCCAAGCCCAAGGACACCCTGATGATCAGCAGAACCCCCGAGGTGACC
TGCGTGGTCGTGGACGTGAGCCACGAGGACCCCGAGGTGAAGTTCAACTGGTACGTGGACG
GCGTGGAGGTGCACAACGCCAAGACCAAGCCTAGAGAGGAGCAGTACAACAGCACCTACA
GAGTGGTGAGCGTGCTGACCGTGCTGCACCAAGACTGGCTGAACGGCAAGGAGTACAAGTG
CAAGGTGAGCAACAAGGCCCTGCCCGCCCCCATCGAGAAGACCATCAGCAAGGCCAAGGGG
CAGCCTAGAGAGCCCCAAGTGTACACCCTGCCCCCTAGCAGAGACGAGCTGACCAAGAACC
AAGTGAGCCTGACCTGCCTGGTGAAGGGCTTCTACCCTAGCGACATCGCCGTGGAGTGGGA
GAGCAACGGGCAGCCCGAGAACAACTACAAGACCACCCCCCCCGTGCTGGACAGCGACGGC
AGCTTCTTCCTGTACAGCAAGCTGACCGTGGACAAGAGCAGATGGCAGCAAGGCAACGTGT
TCAGCTGCAGCGTGATGCACGAGGCCCTGCACAACCACTACACACAGAAGAGCCTGAGCCT
GAGCCCCGGCAAGTAA-3'

LZY3412L

5'-
gccaccATGGACATGAGAGTGCCCGCTCAGCTGCTGGGCCTGCTCCTGCTGTGGCTGCCCGGCG
CCAAGTGCGACATTCAGATGACACAGAGCCCTAGCAGCCTGAGCGCTAGCGTGGGCGACAG
AGTGACCATCACCTGCAGAGCTAGCCAAAGCATCAGCAGCTACCTGAACTGGTATCAGCAGA
AGCCCGGCAAGGCCCCCAAGCTGCTGATCTACGCCGCTAGCAGCCTGCAGAGCGGCGTGCCT
AGCAGATTCAGCGGCAGCGGCAGCGGCACCGACTTCACCCTGACCATCAGCAGCCTGCAACC
CGAGGACTTCGCCACCTACTACTGTCAGCAGAGCTACAGCACCCCTGGCACCTTCGGCCAAG
GCACCAAGGTGGAGATCAAGAGAACCGTGGCCGCCCCTAGCGTGTTCATCTTCCCCCCTAGC
GACGAGCAGCTGAAGAGCGGCACCGCTAGCGTGGTGTGCCTGCTGAACAACTTCTACCCTAG
AGAGGCCAAGGTGCAGTGGAAGGTGGACAACGCCCTGCAGAGCGGCAACAGCCAAGAGAG
CGTGACCGAGCAAGACAGCAAGGACAGCACCTACAGCCTGAGCAGCACCCTGACCCTGAGC
AAGGCCGACTACGAGAAGCACAAGGTGTACGCCTGCGAGGTGACCCACCAAGGCCTGAGCA
GCCCCGTGACCAAGAGCTTCAACAGAGGCGAGTGCAGCTAA -3'
